# Effect of Cannabidiolic Acid, *N*-*Trans*-Caffeoyltyramine and Cannabisin B from Hemp Seeds on microRNA Expression in Human Neural Cells

**DOI:** 10.3390/cimb44100347

**Published:** 2022-10-21

**Authors:** Armando Di Palo, Chiara Siniscalchi, Giuseppina Crescente, Ilenia De Leo, Antonio Fiorentino, Severina Pacifico, Aniello Russo, Nicoletta Potenza

**Affiliations:** Department of Environmental, Biological, and Pharmaceutical Sciences and Technologies—University of Campania “Luigi Vanvitelli”, 81100 Caserta, Italy

**Keywords:** nutraceuticals, microRNA, neural cell, miRNome, Alzheimer’s disease

## Abstract

Given the increasing interest in bioactive dietary components that can modulate gene expression enhancing human health, three metabolites isolated from hemp seeds—cannabidiolic acid, *N*-*trans*-caffeoyltyramine, and cannabisin B—were examined for their ability to change the expression levels of microRNAs in human neural cells. To this end, cultured SH-SY5Y cells were treated with the three compounds and their microRNA content was characterized by next-generation small RNA sequencing. As a result, 31 microRNAs underwent major expression changes, being at least doubled or halved by the treatments. A computational analysis of the biological pathways affected by these microRNAs then showed that some are implicated in neural functions, such as axon guidance, hippocampal signaling, and neurotrophin signaling. Of these, miR-708-5p, miR-181a-5p, miR-190a-5p, miR-199a-5p, and miR-143-3p are known to be involved in Alzheimer’s disease and their expression changes are expected to ameliorate neural function. Overall, these results provide new insights into the mechanism of action of hemp seed metabolites and encourage further studies to gain a better understanding of their biological effects on the central nervous system.

## 1. Introduction

Alzheimer’s disease (AD) is a progressive neurodegenerative disorder leading to cerebral atrophy and dementia. It is biochemically characterized by the neural deposition of amyloid β (Aβ) peptide as extracellular plaques and by the aggregation of hyperphosphorylated Tau protein as intracellular neurofibrillary tangles (NFTs) [1]. Aβ peptide derives from the aberrant proteolytic cleavage of amyloid precursor protein (APP) by secretases, leading to excess amyloid accumulation. Several microRNAs (miRNAs) are implicated in AD [2,3]. Some of these affect Aβ deposition by modulating the expression of APP and secretases, whereas other microRNAs have an impact on NFTs by regulating the expression levels of Tau protein and the kinases/phosphatases acting on them. Furthermore, there are miRNAs implicated in AD for their ability to modulate associated biological processes, such as neuroinflammation, apoptosis, synaptic plasticity, and autophagy.

Several studies have shown that phenols, polyphenols, flavonoids, and other secondary metabolites of plants have anti-aging and cognition-enhancing properties that may ameliorate neural function in AD [4,5,6]. Of these, investigations on *Cannabis sativa* have revealed a marked neuroprotective action of cannabinoids with promising effects in reducing amyloid plaque deposition and stimulating hippocampal neurogenesis [7,8,9]. Furthermore, hydroxycinnamic acid amides from *Cannabis sativa* fruits, and their lignanamide derivatives, have been reported to exert neuroprotective effects. In particular, *N-trans*-caffeoyltyramine showed antioxidant activity against the H_2_O_2_-induced apoptosis in PC12 cells [10], whereas *N-trans*-feruloyltyramine, also known for its antioxidant, anti-inflammatory, anti-melanogenesis, and anticancer activities [11], induced abrogation of Aβ-mediated generation of reactive oxygen species [12].

Several phytochemicals, such as curcumin, quercetin, resveratrol, betulinic acid, capsaicin, epigallocatechin gallate, genistein, and ellagic acid, can modulate microRNA expression in mammalian cells, possibly exerting their health-promoting effects by recruiting these modulators of gene expression [13,14,15,16,17]. In this regard, the term “Nutrigenomics” has recently been introduced to define a field of study that investigates the relationship between bioactive dietary components and human gene expression [18].

Hemp seed is an interesting aliment both for its high content of essential polyunsaturated fatty acids [19] and for the presence of specialized metabolites belonging to precannabinoid and polyphenol classes [20,21]. Based on the recognized ability of phytochemicals to affect human gene expression, we studied the effect of cannabidiolic acid (CBDA), the main phytocannabinoid in hemp seeds, and of *N-trans*-caffeoyltyramine and cannabisin B, which are isolated from defatted hemp seeds, on the miRNome of cultured human neural cells, to evaluate any changes in the expression of AD-related miRNAs.

## 2. Materials and Methods

### 2.1. Isolation of Pure Compounds from Hemp Seeds

Hemp seeds (*Cannabis sativa* cv. Futura 75) underwent ultrasound-assisted maceration with a Branson UltrasonicsTM BransonicTM M3800-E operated in sweep-frequency mode at 40 kHz. *n*-hexane and methanol were used sequentially as extracting solvents at a drug/solvent ratio of 1:5 (g:mL). *n*-hexane extract was fractionated according to Formato et al. [22] to obtain pure cannabidiolic acid (CBDA). The methanol extract was dried with a rotary evaporator, solubilized with water, and partitioned through discontinuous liquid/liquid extraction using the extractant solution ethyl acetate:acetone (2:1, v:v). The organic phase was then fractionated by C18 reversed-phase column chromatography, followed by preparative thin-layer chromatography with a precoated silica gel 60 F_254_ (20 cm × 20 cm, 2 mm, Merck, Darmstadt, Germany). The organic lower phase of a biphasic CHCl_3_:MeOH:H_2_O (13:7:3, v:v:v) solution served as the mobile phase, leading to the purification of *N*-*trans*-caffeoyltyramine and cannabisin. Their identity was initially assessed by the Shimadzu NEXERA UHPLC system using an Omega Luna C18 column (50 mm × 2.1 mm, 1.6 μm) with reference to their relative pure commercial compounds (N-*trans*-caffeoyltyramine, SMB00208, Sigma-Aldrich; cannabisin B, CFN95268, ChemFaces). Then, MS analysis was carried out using AB SCIEXTripleTOF^®^4600 (AB Sciex, Concord, ON, Canada) equipped with a DuoSprayTM ion source, which was operated in the negative electrospray ionization mode. The Q-TOF high-resolution mass spectrometry method involved a full scan TOF survey (dwell time 300 ms, 150–800 Da) and eight information-dependent acquisition MS/MS scans (dwell time 50 ms, 100–750 Da). The MS parameters were as follows: curtain gas (CUR) 35 psi, nebulizer gas (GS 1) 60 psi, heated gas (GS 2) 60 psi, ion spray voltage (ISVF) 4.5 kV, interface heater temperature (TEM) 600 °C, declustering potential (DP) −80 V. The applied collision energy was −45 V with a collision energy spread of 15 V. Analyst^®^ TF 1.7 software controlled the instrument, while PeakView^®^ software Version 2.2 provided data processing.

### 2.2. Cell Cultures

Human SH-SY5Y (SH) cells were grown in Dulbecco’s modified Eagle’s medium with high glucose supplemented with 10% fetal bovine serum (FBS), 50 U/mL penicillin, and 100 μg/mL streptomycin, at 37 °C in a humidified atmosphere containing 5% CO_2_. Cell treatments for the RNA sequencing experiments were performed by incubating SH cells with cannabis compounds at their relative IC_50_ concentrations, as determined by the MTT assay, in FBS-free culture medium for 48 h.

### 2.3. MTT Assay

Cells were seeded in 96-multiwell plates at a density of 1.5 × 10^4^ cells/well. After 24 h of incubation, cells were treated with CBDA at four dose levels (2.5, 5, 10, and 25 μM), and with *N*-*trans*-caffeoyltyramine and cannabisin B at seven concentrations (2.5, 5, 10, 25, 50, 100, and 200 μM). After 48 h of incubation, cells were treated with 150 µL of 0.5 mg/mL 3-(4,5-dimethyl-2-thiazolyl)-2,5-diphenyl-2H-tetrazolium (MTT), having previously been dissolved in FBS-free culture medium for 4 h at 37 °C in a 5% CO_2_ humidified atmosphere. The MTT solution was then removed and 100 µL of DMSO was added to dissolve the produced formazan dye. Finally, the absorbance at 570 nm of each well was determined using a Victor3 Perkin Elmer absorbance reader. Cell viability was expressed as a percentage of mitochondrial redox activity of the cells treated with pure compounds compared to the untreated control. The IC_50_ value of each compound was determined from the relative dose–response curve.

### 2.4. RNA Isolation, Sequencing and Data Analysis

Total RNA purification from cell cultures was performed by miRNeasy Mini Kit (Qiagen) according to the manufacturer’s protocol. RNA concentration was determined with a NanoDropOne spectrophotometer (Thermo Fisher, Waltham, MA, USA) and its quality was assessed with the TapeStation 4200 (Agilent Technologies, Santa Clara, CA, USA). Indexed libraries were prepared from 500 ng/each purified RNA using the NEXTFLEX Small RNA-Seq Kit v3 (PerkinElmer). Libraries were quantified with the TapeStation 4200 (Agilent Technologies) and Qubit fluorometer (Invitrogen Co.), then pooled such that each index-tagged sample was present in equimolar amounts. The pooled samples were then subjected to cluster generation and sequencing using an Illumina NextSeq 550 Dx System (Illumina) in a 1 × 75 single-end format. The generated raw sequence files (.fastq files) underwent quality control analysis using FastQC (http://www.bioinformatics.babraham.ac.uk/projects/fastqc/) (accessed on 22 September 2022). The sRNAbench tool [23] was then used to remove adapter sequences and low-quality reads to obtain the miRNA expression profiles. Finally, the Bioconductor package DESeq2 [24] in R was used to normalize the data using the median of ratios method, and to perform the differential expression analysis between the various experimental condition.

## 3. Results

### 3.1. Isolation of Bioactive Compounds from Hemp Seeds

Cannabidiolic acid (CBDA) is the most abundant precannabinoid in hemp seed and its ability to modulate the release of proinflammatory cytokines and chemokines mediators has recently been evaluated in HaCat cells [25]. Moreover, a hemp seed mixture mainly consisting of phenylamides and lignanamides has been shown to negatively affect U-87 glioblastoma cell line survival and migration [26]. Of these, two structurally related compounds, *N-trans*-caffeoyltyramine and cannabisin B, have also raised interest because of their antioxidant activity [10].

**Figure 1:**
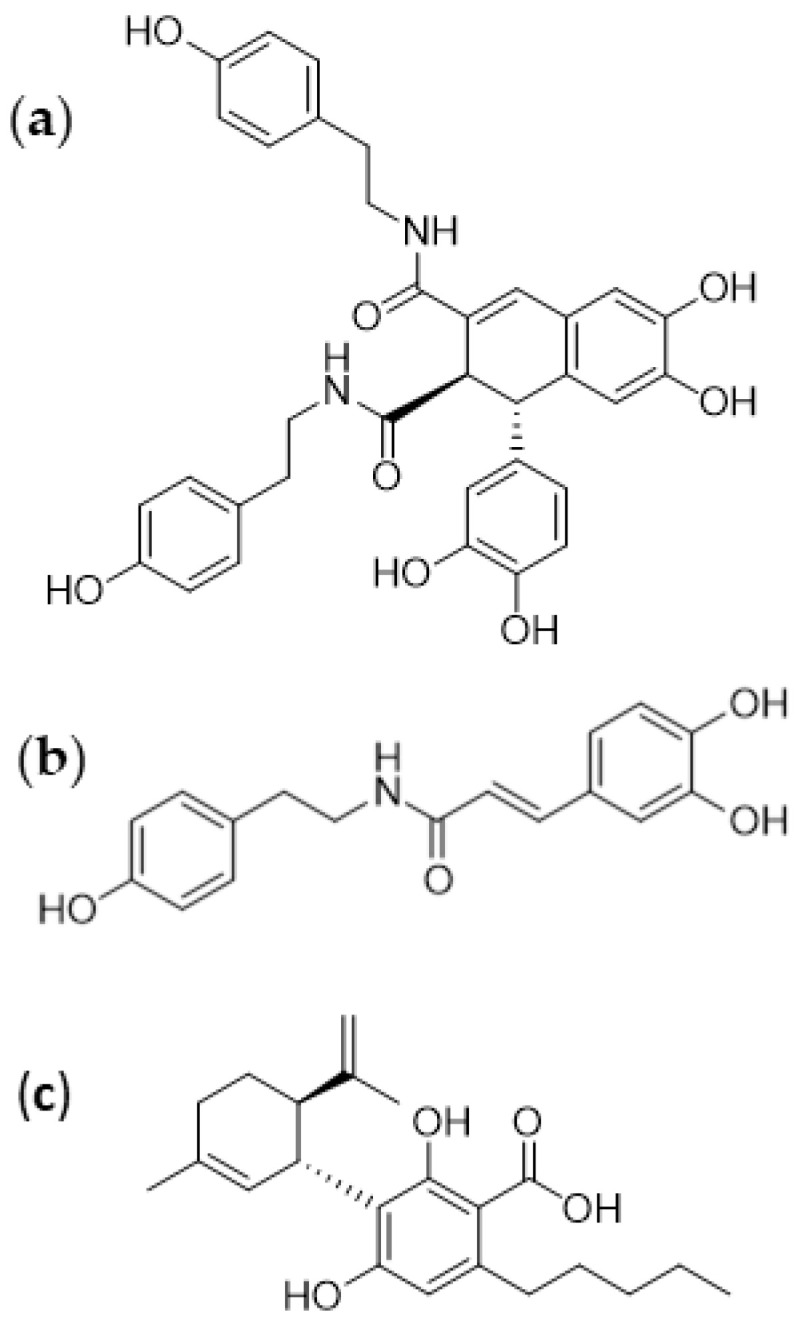
Chemical structures of the three metabolites isolated from hemp seeds. Cannabisin B (**a**), *N*-*trans*-caffeoyltyramine (**b**), and cannabidiolic acid (**c**) were isolated from *Cannabis sativa* seeds.

In this work, cannabidiolic acid was purified according to an established procedure based on fractionation of an n-hexane extract of hemp seeds [22], whereas two other major metabolites, *N-trans*-caffeoyltyramine and cannabisin B (Figure 1), were obtained by an optimized procedure based on the C18 reversed-phase column chromatography of a methanol extract of hemp seeds.

**Figure 2:**
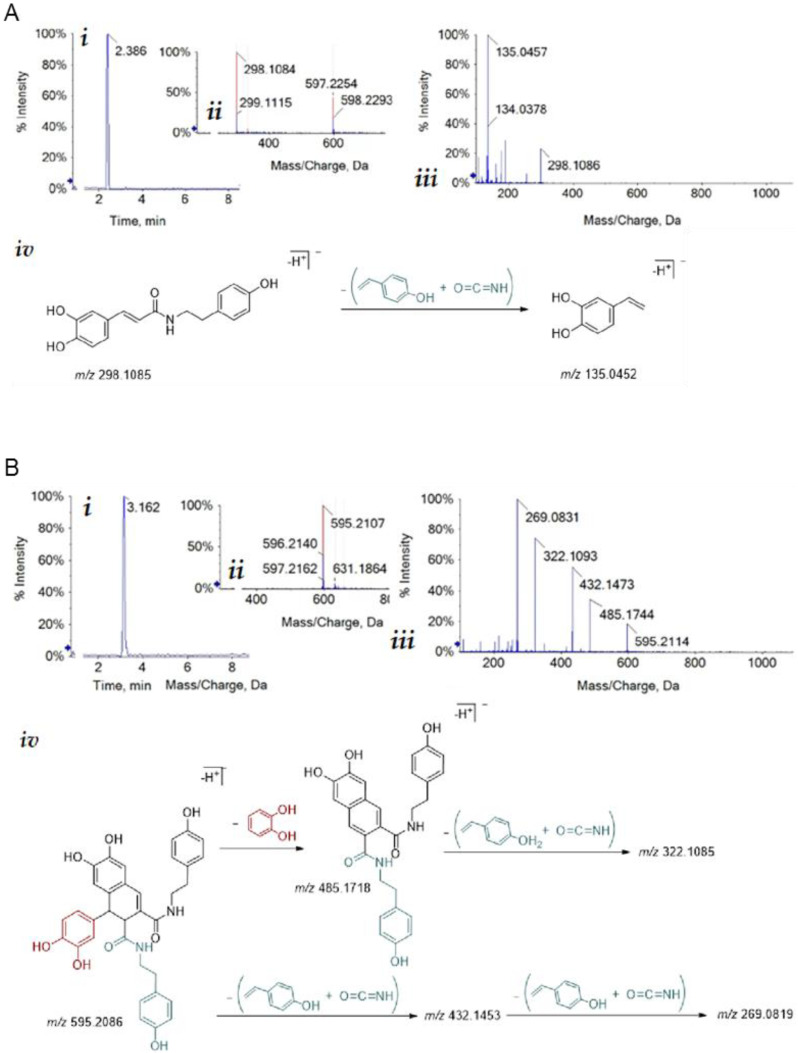
Structural characterization of hemp seed metabolites by mass spectrometry. (**A**) *N-trans*-caffeoyltyramine (i) Total Ion Chromatogram (TIC); (ii) TOF-MS spectrum showing the [M-H]^-^ and [2M-H]^-^ ions at *m/z* 298.1084 and 597.2254, respectively; (iii) TOF-MS/MS of the [M-H]^-^ ion; (iv) proposed fragmentation pattern; theoretical *m/z* value is below each chemical structure. (**B**) Cannabisin B (i) Total Ion Chromatogram (TIC); (ii) TOF-MS spectrum showing the [M-H]^-^ ion at *m/z* 595.2107; (iii) TOF-MS/MS of the [M-H]^-^ ion; (iv) proposed fragmentation pattern; theoretical *m/z* value is below each chemical structure.

These major metabolites were identified by comparing their Ultraviolet Diode Array Detection (UV-DAD) and TOF-MS/MS spectra with those of pure commercial reference compounds (Figure 2). Briefly, according to the findings of Nigro et al. [26], the TOF-MS spectrum of *N-trans*-caffeoyltyramine showed the peak of deprotonated molecular ion at *m/z* 298.1084 and its fragmentation yielded the TOF-MS2 base peak at *m/z* 135.0457, as expected for 4-vinylcatecholate. The deprotonated molecular ion of cannabisin B at *m/z* 595.2107 provided, in the TOF-MS/MS experiment, the fragment ion at *m/z* 485.1744, due to the loss of a catechol unit at *m/z* 432.1473, which was the characteristic lignanamide loss of isocyanic acid and p-hydroxystyrene. Further loss of isocyanic acid and p-hydroxystyrene yielded fragment ions at *m/z* 269.0831 and *m/z* 322.1093.

### 3.2. Cytotoxicity Assays

**Figure 3:**
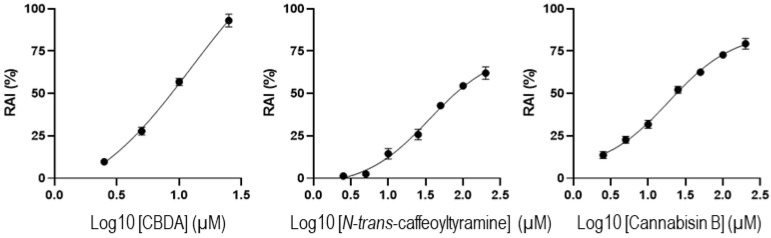
Mitochondrial Redox Activity Inhibition (RAI). SH cells were treated with cannabidiolic acid (CBDA), *N*-*trans*-caffeoyltyramine, or cannabisin B for 48 h, and RAI was assessed by MTT assay. Values are the mean ± SD of three independent experiments performed in triplicate.

The three compounds then underwent cytotoxicity testing towards cultured SH cells by the MTT assay. This was based on the mitochondrial redox activity of live cells that can convert the water-soluble dye MTT into insoluble purple formazan crystals. Measurement of the amount of formazan then gives an estimate of the number of cultured live cells. Through this assay, it was found that CBDA inhibited redox mitochondrial activity in a dose-dependent manner, with an IC_50_ of 8.7 μM (Figure 3). The phenylamide compounds showed a milder toxicity profile with IC_50_ values for *N*-*trans*-caffeoyltyramine and cannabisin B of 59 and 27 μM, respectively.

### 3.3. Hemp Seed Metabolites Change the microRNA Expression Profiles of Neural Cells

**Figure 4:**
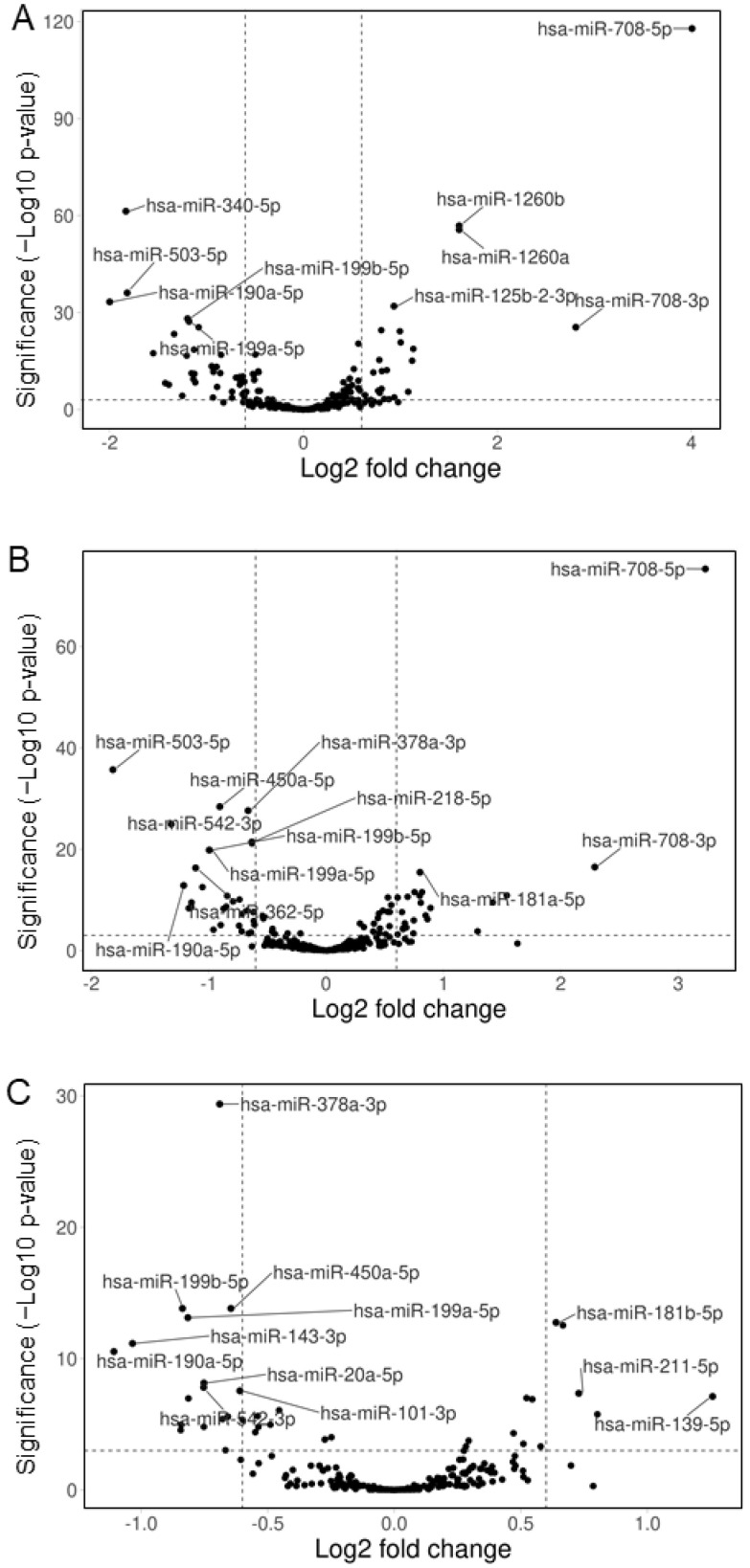
Volcano plots of microRNA expression changes in cultured neural cells. SH cells were treated with cannabisin B (**A**), *N*-*trans*-caffeoyltyramine (**B**), or cannabidiolic acid (**C**) for 48 h, then microRNA expression changes were detected by next-generation small RNA sequencing. Plots were prepared by the program VolcaNoseR [27]. Fields outside the vertical broken lines include miRNAs showing a fold change > 1.5; fields above the horizontal broken line contain miRNAs with a highly significant variation (*p* < 0.001); hsa, homo sapiens.

Cultured human neural SH cells were incubated with three major metabolites isolated from hemp seeds, cannabisin B, *N*-*trans*-caffeoyltyramine, or cannabidiolic acid (CBDA). After 48 h, total RNA was extracted and the microRNA content was characterized by next-generation small RNA sequencing. This was based on the construction of cDNA libraries that underwent high-throughput sequencing, revealing the normalized number of reads for each human microRNA in the sample. A comparison of the three samples with a control of untreated cells revealed large microRNA expression changes, especially with cannabisin B and *N*-*trans*-caffeoyltyramine (Figure 4).

**Figure 5:**
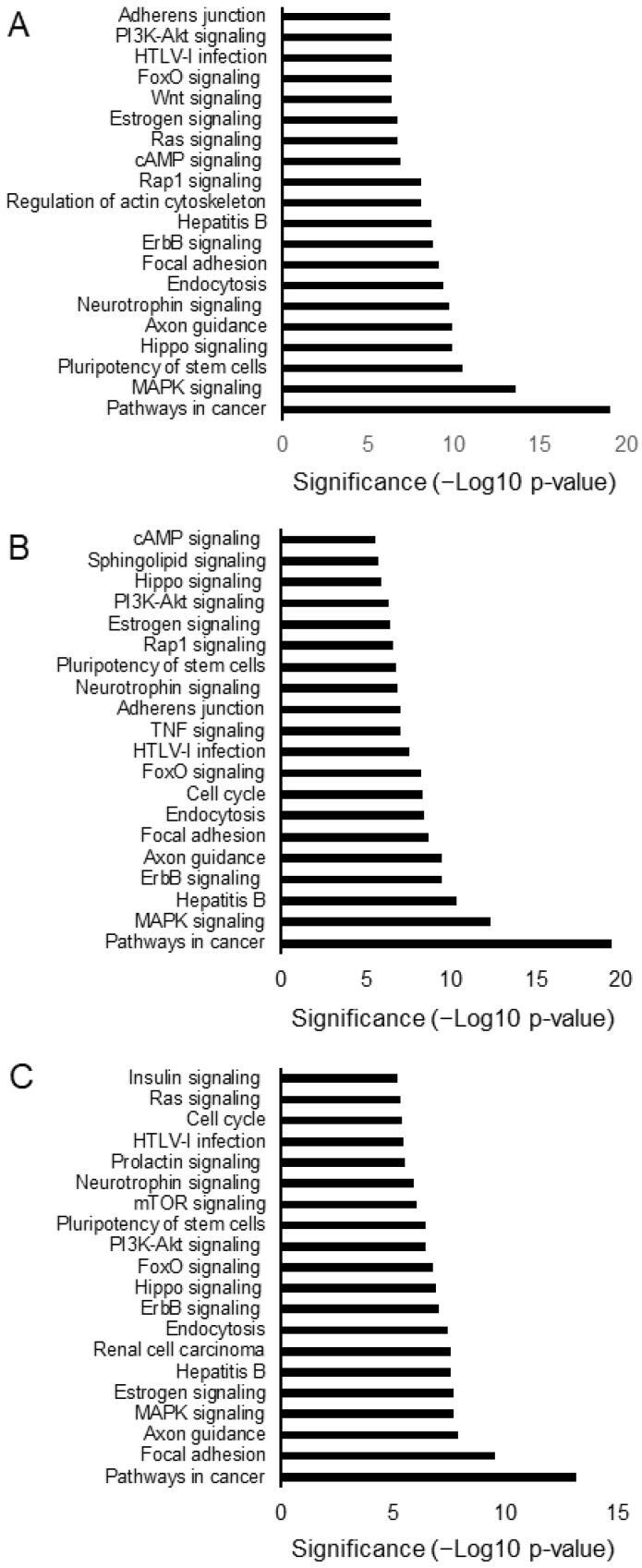
Biological pathways potentially affected by a miRNA-based mechanism. The whole targetomes (predicted and validated) of microRNAs deregulated by cannabisin B (**A**), *N*-*trans*-caffeoyltyramine (**B**), and cannabidiolic acid (**C**) were analyzed by the program DAVID and the top-20 significantly enriched KEGG pathways were displayed. Biological pathways were considered statistically significant if *p*-value was less than 0.05 (Benjamini-Hochberg procedure for multiple correction).

The selection of microRNAs with expression changes of at least 1.5-fold yielded a list of 68, 51, and 21 miRNAs for cannabisin B, *N*-*trans*-caffeoyltyramine, and CBDA, respectively (Supplementary Tables S1–S3). The biological pathways potentially affected by these miRNAs were then analyzed by considering their whole targetomes through miRWalk platform [28]. In particular, for each miRNA, we considered all the experimentally validated targets reported by the miRTarbase tool and predicted targets identified by both TargetScan and miRDB tools with a cut-off score ≥ 0.5 for miRNA–mRNA pairings, in both the untranslated and coding sequences of target transcripts. Pathway enrichment analysis of target genes was then performed using the program DAVID (https://david.ncifcrf.gov/) (accessed on 1 March 2022); this consisted of an integrated biological knowledgebase and analytic tools aimed at systematically extracting biological meaning from large gene/protein lists [29]. In particular, we focused on the significantly enriched pathways using KEGG as a reference database. This approach revealed that neural functions, such as “Axon guidance”, “Hippo signaling”, and “Neurotrophin signaling” were among the top-ranking pathways predicted to be affected by the de-regulated miRNAs (Figure 5).

### 3.4. Comparison of the Effects of the Three Specialized Hemp Seed Metabolites

**Figure 6:**
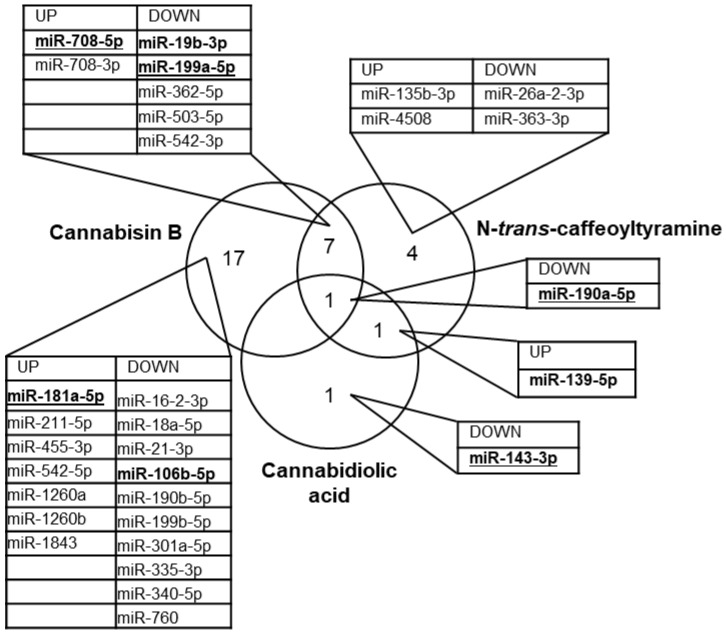
Venn diagram of microRNAs from neural SH cells showing expression levels markedly affected by cannabis compounds. Selection was limited to those microRNAs with an absolute value of log2 fold change ≥ 1. MicroRNAs involved in Alzheimer’s disease are marked in bold; those varying such as to expect an amelioration of neural function in AD are underlined.

To obtain a more concise view of the expression changes, only those miRNAs that showed at least doubled or halved expression were considered, thus retrieving a pool of 31 molecules (Figure 6). Under these conditions, cannabisin B dysregulated 25 miRNAs, with eight showing variations very similar to those observed with *N*-*trans*-caffeoyltyramine treatment. Only five miRNAs among the 13 affected by *N*-*trans*-caffeoyltyramine were not similarly changed by cannabisin B. This similarity between the effects of cannabisin B and *N*-*trans*-caffeoyltyramine is not surprising given the resemblance of their chemical structures, such that both are provided with phenol and catechol moieties (Figure 1). In this regard, it is noteworthy that the most upregulated miRNA, miR-708-5p, showing a 16-fold and 9-fold increase by cannabisin B and *N*-*trans*-caffeoyltyramine, respectively, has also been reported to be strongly upregulated by bisphenol [30].

## 4. Discussion

The most upregulated miRNA revealed by this study, miR-708-5p, appeared to be negatively correlated to AD, since its content in the cerebrospinal fluid of AD patients was found to be markedly lower than in neurologically normal controls [31]. Additionally, miR-199a-5p was affected by the cannabis compounds, being downregulated by cannabisin B and *N*-*trans*-caffeoyltyramine by 56% and 50%, respectively (Supplementary Tables S1 and S2). This miRNA is known to be associated with AD because it targets neuritin, a neurotrophin that is involved in neural development and plasticity and is downregulated in AD [32]. MicroRNA-181a-5p is mainly affected by cannabisin B, which increases its cellular content by 2-fold (supplementary Table S1). This miRNA has previously been shown to play a protective role in AD by targeting FOXO1, a transcription factor involved in insulin signaling and apoptosis. In this regard, lentiviral-mediated expression of miR-181a-5p in the brain of mice ameliorates plaque deposition and cognitive function [33]. MicroRNA 190a-5p is the only miRNA undergoing similar expression changes upon treatment with the three cannabis compounds (Figure 6). Its downregulation may be a desired effect in AD since the content of miR-190a-5p in neural-derived small extracellular vesicles from AD patients is known to be significantly upregulated when compared with controls [34]. The only miRNA specifically modified by CBDA is miR-143-3p, with an expression level that is reduced by 51% (Figure 6). This miRNA is upregulated in the serum of AD patients [35] and a study on a cell model of AD has shown that its downregulation promotes neuronal survival by upregulating its target neuregulin-1, an EGF-like protein playing a crucial role in brain development, neuronal migration, differentiation and synapse formation [36]. With regards to the possible mechanisms of modulation of microRNA expression by the three compounds tested in this study, it should be considered that miRNA precursors are transcribed by RNA Polymerase II. The activity of this enzyme critically depends on the specific combination of transcription factors that are active in the nucleus. Both activators and repressors of transcription may then affect microRNA expression, depending on the biochemical pathways that are activated in response to extracellular signal compounds. Within this network, bioactive compounds may affect microRNA expression by interacting with membrane receptors, thus inducing agonistic or antagonistic effects on the expression of specific genes.

## 5. Conclusions

This study shows that three metabolites from edible hemp seeds, cannabisin B, *N*-*trans*-caffeoyltyramine, and CBDA, can modify the miRNome of cultured human neural cells with effects on specific microRNAs that are implicated in neural functions. This pilot transcriptomic analysis may provide the basis for functional in vivo studies aimed at a direct evaluation of the effects of the three compounds on specific microRNAs and related biochemical pathways. One possible biomolecular approach may involve the oral administration of the three metabolites to mice, followed by the quantitation of miR-708-5p, miR-181a-5p, miR-190a-5p, miR-199a-5p, and miR-143-3p in the brain by real-time qPCR. If their expression changes are confirmed, the neural expression of the genes targeted by these microRNAs, such as neuritin, FOXO1, and neuregulin-1, may be assessed. However, a balanced evaluation of the beneficial effects of the three metabolites on microRNA expression should also take into account their mild cytotoxic activity (Figure 3). In this regard, treatments at low metabolite concentrations might well be conceivable given the large variation in microRNA expression registered in this study.

## Data Availability

Not applicable.

## Supplementary Materials

The following supporting information can be downloaded at: https://www.mdpi.com/article/10.3390/cimb44100347/s1, Table S1: Effect of cannabisin B on miRNA expression in neural SH cells; Table S2: Effect of *N*-*trans*-caffeoyltyramine on miRNA expression in neural SH cells; Table S3: Effect of cannabidiolic acid on miRNA expression in neural SH cells.
