# Supplementary material for: Effect of Cannabidiolic Acid, N-Trans-Caffeoyltyramine and Cannabisin B from Hemp Seeds on microRNA Expression in Human Neural Cells
Source: Curr Issues Mol Biol. 2022 Oct 21;44(10):5106–16. doi: 10.3390/cimb44100347 (PMC9600072; doi:10.3390/cimb44100347)
Supplement: Supplementary file 1 [file cimb-44-00347-s001.zip › cimb-1966157-supplementary.pdf]

Supplementary Table S1. Effect of cannabisin B on miRNA expression in neural SH cells

miRNA	mean of normalized counts	log2 fold change	adjusted p value
hsa-miR-708-5p	653	4,01	1,38E-118
hsa-miR-708-3p	111	2,81	3,51E-26
hsa-miR-1260a	9467	1,61	2,26E-56
hsa-miR-1260b	9475	1,61	1,41E-57
hsa-miR-211-5p	880	1,13	1,72E-19
hsa-miR-542-5p	638	1,12	8,09E-16
hsa-miR-1843	272	1,08	3,32E-06
hsa-miR-455-3p	19117	1,00	1,76E-21
hsa-miR-181a-5p	32243	1,00	6,25E-25
hsa-miR-4508	117	0,98	5,24E-03
hsa-miR-29c-5p	122	0,94	1,74E-04
hsa-miR-125b-2-3p	2453	0,93	1,06E-32
hsa-miR-671-3p	212	0,88	6,64E-04
hsa-miR-126-3p	537	0,86	6,79E-13
hsa-miR-139-5p	181	0,83	5,36E-04
hsa-miR-320a-3p	20799	0,81	1,32E-12
hsa-miR-320b	389	0,81	3,87E-07
hsa-miR-146a-5p	29016	0,80	2,91E-25
hsa-miR-500a-3p	240	0,79	7,23E-06
hsa-miR-320c	129	0,78	7,07E-03
hsa-miR-34b-3p	2242	0,78	4,65E-16
hsa-miR-135b-3p	255	0,73	2,94E-03
hsa-miR-339-3p	769	0,72	3,41E-12
hsa-miR-150-5p	184	0,69	1,01E-03
hsa-miR-4510	222	0,65	1,81E-03
hsa-miR-125a-3p	706	0,64	3,58E-05
hsa-miR-5100	456	0,63	8,00E-03
hsa-miR-424-5p	3671	-0,59	3,28E-06

hsa-miR-26a-2-3p	262	-0,59	4,25E-03
hsa-miR-335-5p	590	-0,60	9,53E-04
hsa-miR-3065-5p	485	-0,60	5,08E-06
hsa-miR-143-3p	960	-0,60	7,43E-05
hsa-miR-101-3p	7304	-0,61	2,00E-09
hsa-miR-874-3p	405	-0,62	2,87E-03
hsa-miR-98-3p	222	-0,62	5,86E-03
hsa-miR-107	4329	-0,62	5,99E-11
hsa-miR-363-3p	566	-0,63	1,80E-05
hsa-let-7a-3p	2254	-0,65	4,74E-09
hsa-miR-199b-3p	24702	-0,65	9,27E-11
hsa-miR-17-5p	3605	-0,66	2,15E-08
hsa-miR-199a-3p	24888	-0,66	8,03E-11
hsa-miR-532-5p	2406	-0,70	1,36E-10
hsa-miR-15a-5p	2556	-0,74	3,04E-06
hsa-let-7f-1-3p	255	-0,74	2,40E-04
hsa-miR-193b-3p	169	-0,82	6,74E-03
hsa-miR-15b-5p	25706	-0,85	1,17E-17
hsa-miR-20a-5p	2993	-0,86	5,55E-12
hsa-miR-362-3p	449	-0,89	1,03E-07
hsa-miR-15b-3p	1629	-0,90	6,19E-14
hsa-miR-449a	664	-0,93	2,27E-12
hsa-miR-301a-3p	345	-0,93	1,97E-04
hsa-miR-7-5p	9260	-0,95	4,00E-14
hsa-miR-106b-5p	7756	-1,08	3,63E-26
hsa-miR-21-3p	661	-1,12	8,76E-12
hsa-miR-542-3p	3553	-1,13	2,95E-19
hsa-miR-19b-3p	660	-1,13	3,78E-10
hsa-miR-301a-5p	268	-1,15	6,07E-12
hsa-miR-199a-5p	1028	-1,18	3,97E-28
hsa-miR-199b-5p	993	-1,20	7,85E-29
hsa-miR-18a-5p	517	-1,20	2,10E-17

hsa-miR-190b-5p	123	-1,25	5,43E-05
hsa-miR-362-5p	1225	-1,33	4,33E-24
hsa-miR-760	459	-1,39	2,12E-08
hsa-miR-335-3p	118	-1,42	6,60E-09
hsa-miR-16-2-3p	221	-1,55	3,87E-18
hsa-miR-503-5p	4633	-1,82	8,17E-37
hsa-miR-340-5p	6167	-1,83	4,60E-62
hsa-miR-190a-5p	718	-2,00	4,98E-34

Selection was limited to those microRNAs showing normalized counts ≥ 100, absolute value of log2 fold change ≥ 0.59, and Benjamini-Hochberg adjusted p value < 0.01.

Supplementary Table S2. Effect of *N-trans*-caffeoyltyramine on miRNA expression in neural SH cells

miRNA	mean of normalized counts	log2 fold change	adjusted p value
hsa-miR-708-5p	653	3,23	4,57E-76
hsa-miR-708-3p	111	2,29	3,25E-17
hsa-miR-135b-3p	255	1,54	1,31E-11
hsa-miR-139-5p	181	1,42	3,12E-10
hsa-miR-4508	117	1,29	1,56E-04
hsa-miR-194-5p	578	0,89	3,85E-09
hsa-miR-500a-3p	240	0,86	6,59E-07
hsa-miR-1249-3p	1125	0,85	1,15E-07
hsa-miR-211-5p	880	0,81	3,68E-10
hsa-miR-181a-5p	32260	0,8	3,53E-16
hsa-miR-192-5p	799	0,8	1,59E-11
hsa-miR-455-3p	19117	0,75	2,91E-12
hsa-miR-132-3p	451	0,75	1,08E-06
hsa-miR-150-5p	184	0,74	4,13E-04
hsa-miR-339-3p	769	0,69	2,43E-11
hsa-miR-320b	410	0,68	2,82E-05
hsa-miR-542-5p	638	0,67	4,09E-06
hsa-miR-200c-3p	181	0,67	4,71E-03
hsa-miR-502-3p	318	0,66	4,22E-05
hsa-miR-320a-3p	20799	0,65	2,57E-08
hsa-miR-135b-5p	37964	0,6	3,37E-11
hsa-miR-23c	262	0,6	6,45E-04
hsa-miR-449°	664	-0,6	7,68E-06
hsa-let-7a-3p	2241	-0,62	2,34E-08
hsa-miR-20a-5p	2993	-0,62	1,40E-06
hsa-miR-218-5p	11811	-0,63	3,67E-22
hsa-miR-16-2-3p	221	-0,64	2,60E-04
hsa-miR-378a-3p	9224	-0,66	2,39E-28

hsa-miR-106a-5p	449	-0,66	4,13E-04
hsa-miR-17-5p	3605	-0,67	1,33E-08
hsa-miR-125b-1-3p	6965	-0,72	6,11E-08
hsa-miR-222-5p	390	-0,72	1,67E-04
hsa-miR-340-5p	6167	-0,74	8,32E-11
hsa-miR-362-3p	449	-0,74	1,24E-05
hsa-miR-92b-3p	1848	-0,79	2,05E-10
hsa-miR-424-5p	3671	-0,84	1,60E-11
hsa-miR-18a-5p	517	-0,85	2,11E-09
hsa-miR-143-3p	960	-0,87	5,52E-09
hsa-miR-874-3p	405	-0,9	9,21E-06
hsa-miR-450a-5p	18778	-0,91	4,20E-29
hsa-miR-335-3p	118	-0,96	7,72E-05
hsa-miR-450a-1-3p	100	-0,97	1,29E-03
hsa-miR-199b-5p	993	-0,99	1,46E-20
hsa-miR-199a-5p	1029	-1	1,72E-20
hsa-miR-363-3p	566	-1,05	3,01E-13
hsa-miR-362-5p	1225	-1,11	4,85E-17
hsa-miR-19b-3p	624	-1,15	1,82E-09
hsa-miR-26a-2-3p	262	-1,17	4,54E-09
hsa-miR-190a-5p	718	-1,21	1,40E-13
hsa-miR-542-3p	3553	-1,32	1,13E-25
hsa-miR-503-5p	4633	-1,82	1,98E-36

Selection was limited to those microRNAs showing normalized counts ≥ 100, absolute value of log2 fold change ≥ 0.59, and Benjamini-Hochberg adjusted p value < 0.01.

Supplementary Table S3. Effect of cannabidiolic acid on miRNA expression in neural SH cells

miRNA	mean of normalized counts	log2 fold change	adjusted p value
hsa-miR-139-5p	181	1,26	7,60E-08
hsa-miR-24-1-5p	491	0,80	1,73E-06
hsa-miR-211-5p	880	0,73	4,38E-08
hsa-miR-181b-5p	11293	0,67	2,94E-13
hsa-miR-101-3p	7219	-0,59	2,83E-08
hsa-miR-92b-3p	1848	-0,60	4,60E-06
hsa-miR-378a-5p	253	-0,61	4,99E-03
hsa-miR-450a-5p	18778	-0,65	1,52E-14
hsa-miR-362-5p	1225	-0,66	2,60E-06
hsa-miR-378c	291	-0,67	9,39E-04
hsa-miR-18a-5p	517	-0,68	4,05E-06
hsa-miR-378a-3p	9224	-0,69	4,24E-30
hsa-miR-20a-5p	2993	-0,75	7,21E-09
hsa-miR-362-3p	449	-0,75	1,55E-05
hsa-miR-542-3p	3553	-0,75	1,53E-08
hsa-miR-503-5p	4633	-0,81	1,07E-07
hsa-miR-199a-5p	1029	-0,82	7,62E-14
hsa-miR-199b-5p	993	-0,84	1,52E-14
hsa-miR-19b-3p	660	-0,84	1,08E-05
hsa-miR-143-3p	960	-1,03	7,01E-12
hsa-miR-190a-5p	718	-1,11	2,91E-11

Selection was limited to those microRNAs showing normalized counts ≥ 100, absolute value of log2 fold change ≥ 0.59, and Benjamini-Hochberg adjusted p value < 0.01.
